# Supplementary material for: Soil bacterial networks are less stable under drought than fungal networks
Source: Nat Commun. 2018 Aug 2;9:3033. doi: 10.1038/s41467-018-05516-7 (PMC6072794; doi:10.1038/s41467-018-05516-7)
Supplement: Supplementary file 1 — Supplementary Information [file 41467_2018_5516_MOESM1_ESM.pdf]

**Supplementary Information**

# Soil bacterial networks are less stable under drought than fungal networks

De Vries et al.

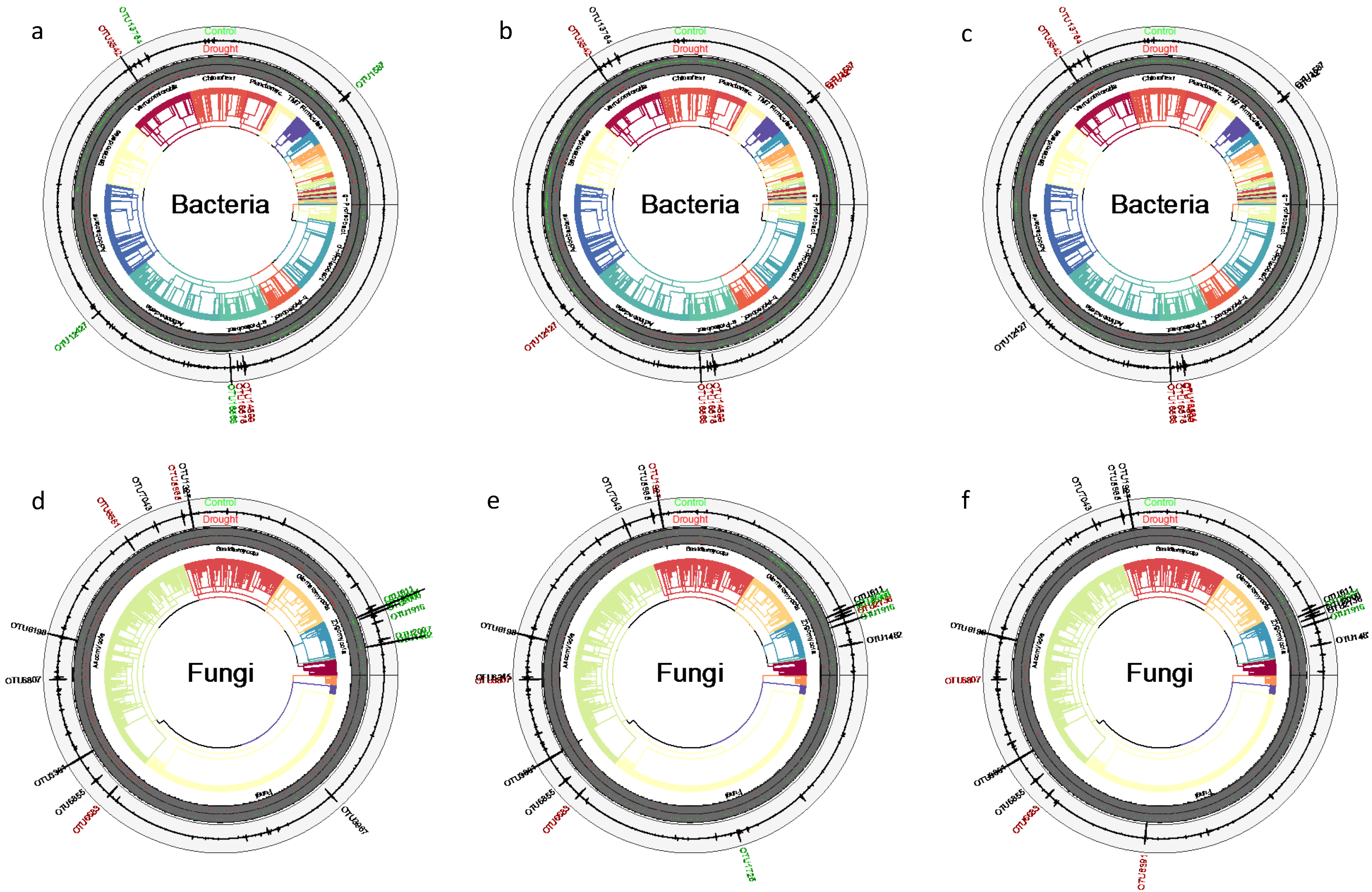
a
b
c
d
e
f
Control
Drought
Bacteria
Fungi
Verrucomicrobia
Chloroflexi
Planctomyc
TM7
Firmicutes
Bacteroidetes
Acidobacteria
Actinobacteria
Basidiomycota
Glomeromycota
Zygomycota
Ascomycota
OTU3542
OTU13784
OTU1587
OTU12427
OTU6198
OTU5807
OTU7043
OTU6855
OTU5611
OTU1916
OTU1482
OTU3867

Supplementary Figure 1. Bacterial (a-c) and fungal (d-f) community response to drought over time. The inner ring represents the taxonomic tree (coloured by phylum, with phyla labelled only if > 100 representative OTUs). The middle ring displays indicator OTUs that showed a significant response to drought, with central points (black) identifying non responsive taxa, inner points (red) OTUs elevated in abundance in droughted (a, d) or rewetted soils (b, e & c, f), and outermost points (green) denoting OTU's that are at greater abundance in control soils. The outer ring represents OTU average abundance in drought and control (y limits = -3 to 3 %), with lines pointing inward representing the average abundance across drought treatments, and lines pointing outward representing the average abundance in control treatments. Indicator OTU identities are given for dominant taxa (> 1% abundance) outside the outer ring coloured as before to indicate those not affected (black), enriched (red), or reduced (green) by drought. For further information on the identities of all indicators see Suppl. Datafile 1. a, d = end of drought sampling; b, e = early recovery sampling; c, f = late recovery sampling.

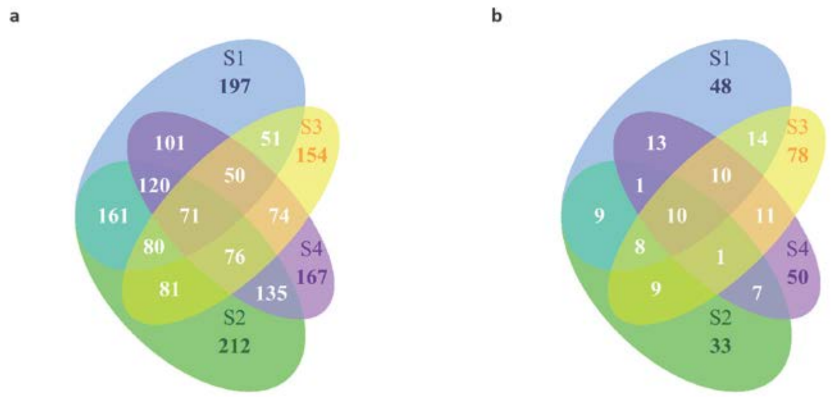


Supplementary Figure 2. Overlap in network membership in bacterial (a) and fungal (b) control networks over time (S1 = before drought sampling, S2 = end of drought sampling, S3 = early recovery sampling, S4 = late recovery sampling). Bacterial control networks had a higher proportion of shared OTUs between sampling dates. Shared proportion of OTUs between S1 and S2 was 0.32 and 0.17 for bacterial and fungal networks, respectively (Chi-squared = 15.02, df = 1, $P$ = 0.0001); between S2 and S3 was 0.24 and 0.15 for bacterial and fungal networks, respectively (Chi-squared = 8.23, df = 1, $P$ = 0.004), and between S3 and S4 was 0.23 and 0.15 for bacterial and fungal networks, respectively (Chi-squared = 6.65, df = 1, $P$ = 0.009).

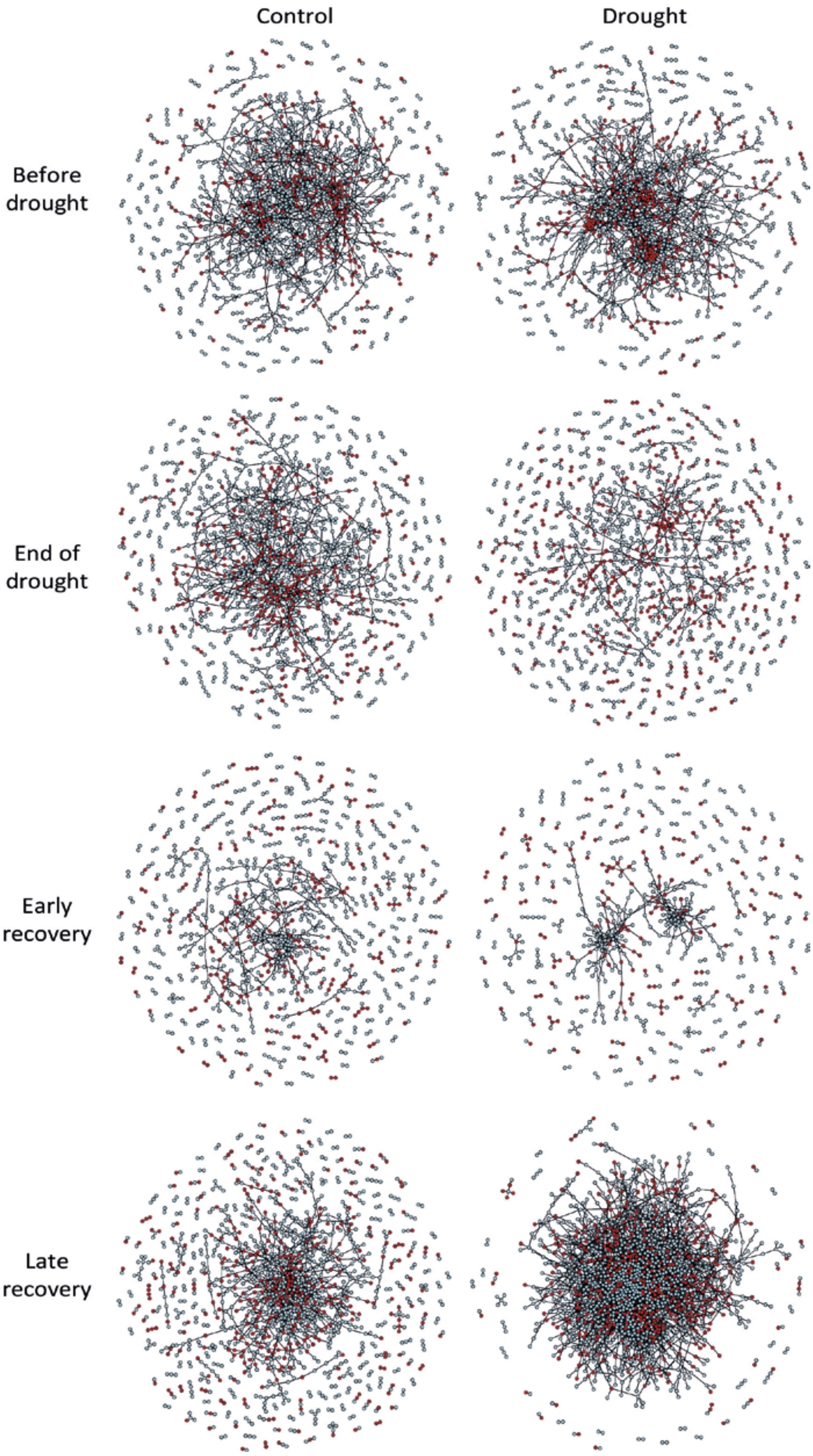


Supplementary Figure 3. Combined bacterial-fungal co-occurrence networks over time as affected by drought. Nodes represent individual OTUs; edges represent significant positive Spearman correlations (rho > 0.6). Red nodes are fungal OTUs, blue nodes are bacterial OTUs. For more network properties see Suppl. Table 1.

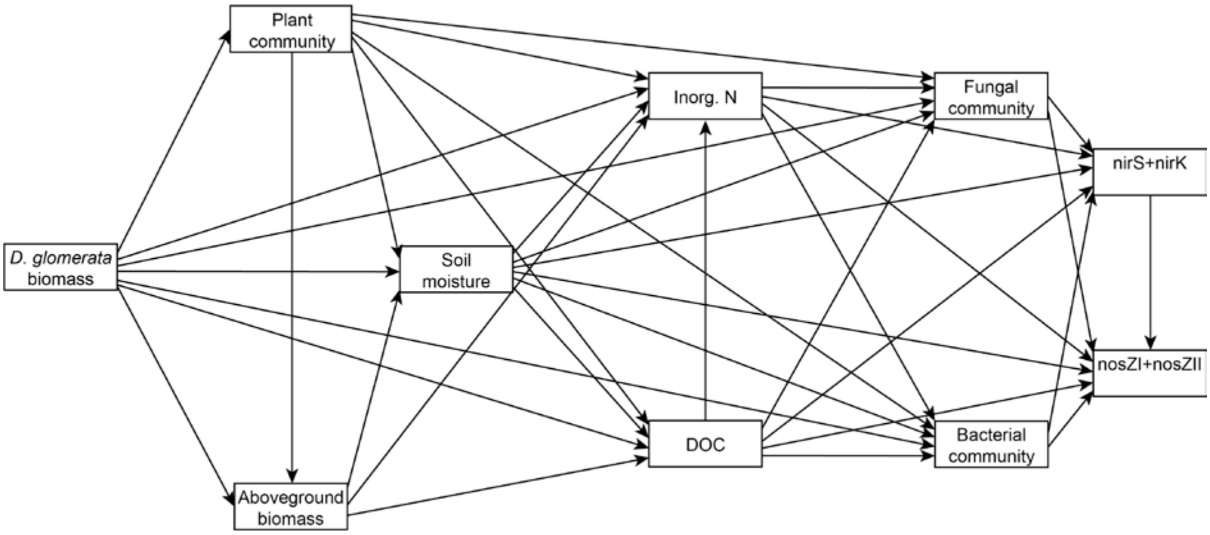


Supplementary Figure 4: *A priori* Structural Equation Model testing the relationships between plant community properties, soil moisture and N and C availability, and microbial community properties at the final, late recovery sampling. See Supplementary Note 1 for explanations.

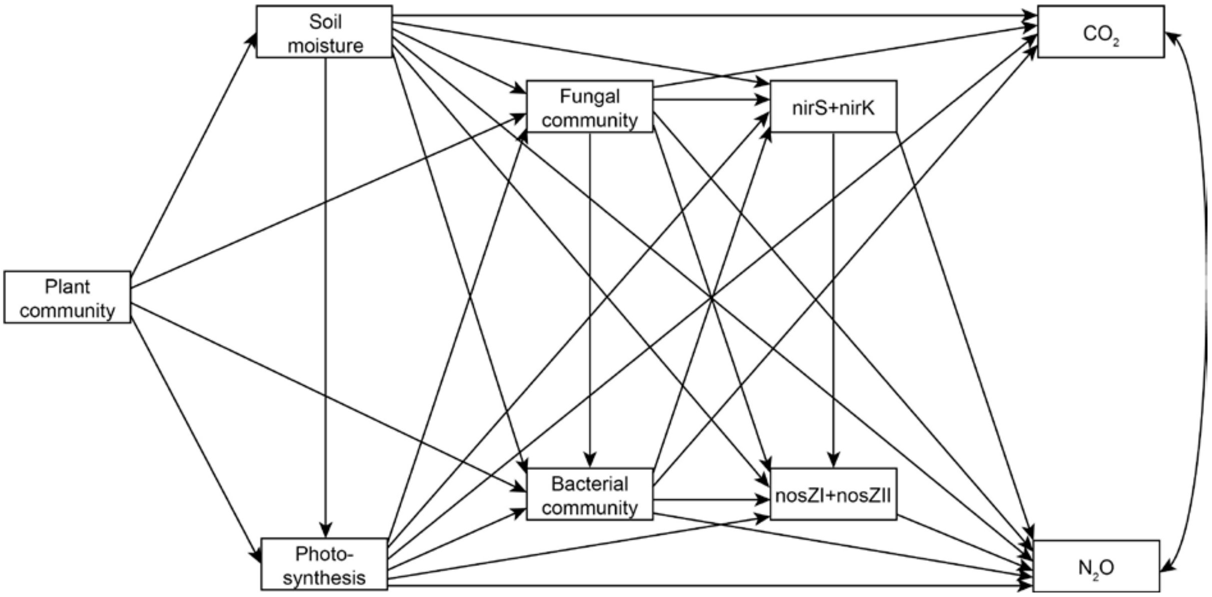


Supplementary Figure 5. *A priori* repeated measures (multi-group) SEM testing the relationships between plant communities, microbial community properties, and $CO_2$ and $N_2O$ fluxes. See Supplementary Note 2 for explanations.

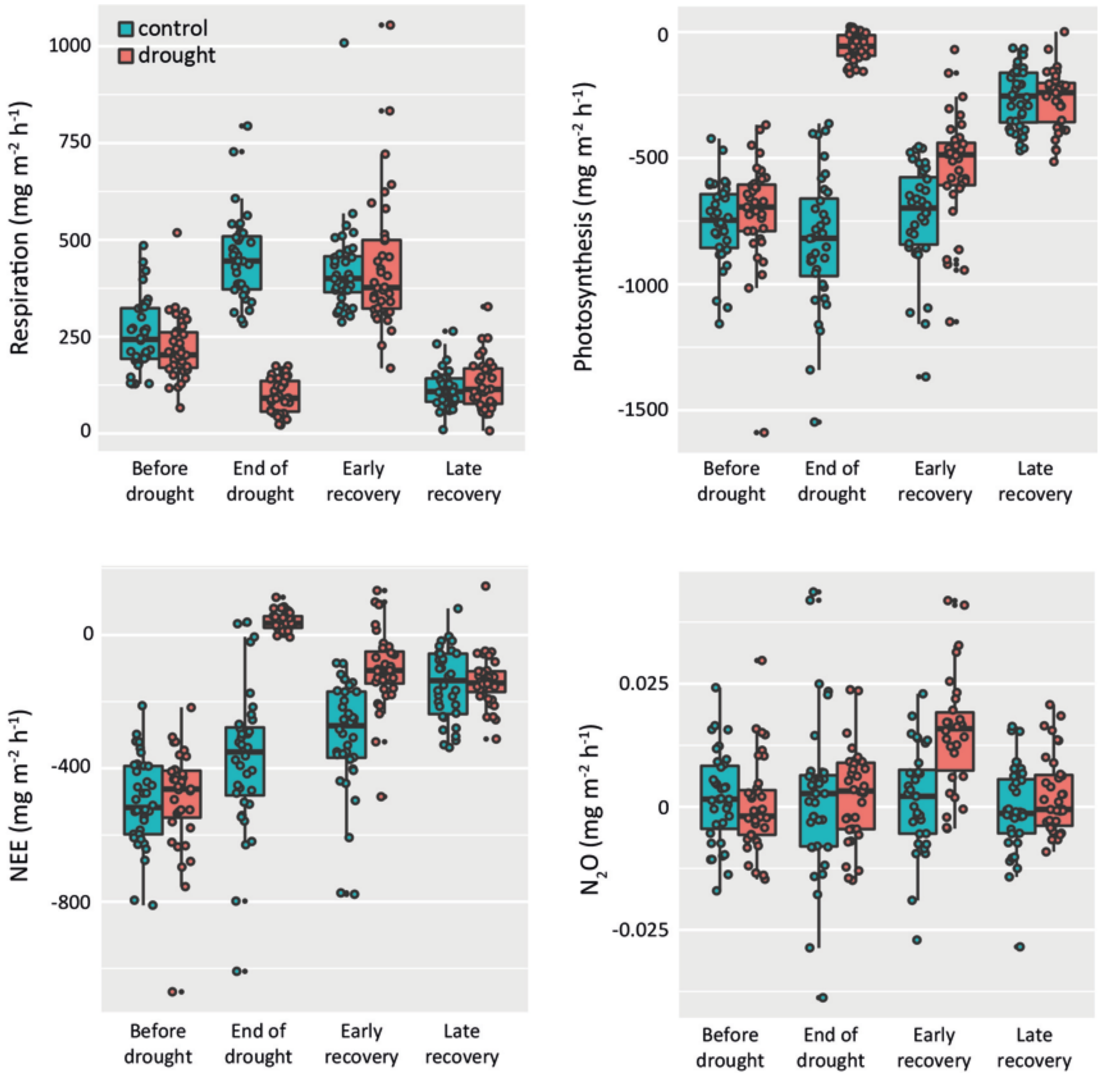


Supplementary Figure 6. The response of ecosystem $CO_2$, and $N_2O$ fluxes to drought over the duration of the experiment. The effect of drought on ecosystem respiration, photosynthesis, and net ecosystem exchange (NEE) varied over time (panel a-c: repeated measures ANOVA Sampling x Drought interaction $F_{3,201} = 72.4$, $P < 0.001$, $F_{3,201} = 71.5$, $P < 0.001$, and $F_{3,201} = 37.9$, $P < 0.001$, respectively). The effect of drought on $N_2O$ production also varied over time (panel d: repeated measures ANOVA Sampling x Drought interaction $F_{3,188} = 6.1$, $P < 0.001$). Lines in boxes represent median, top and bottom of boxes represent first and third quartiles, and whiskers represent 1.5 inter quartile range; dots represent single observations.

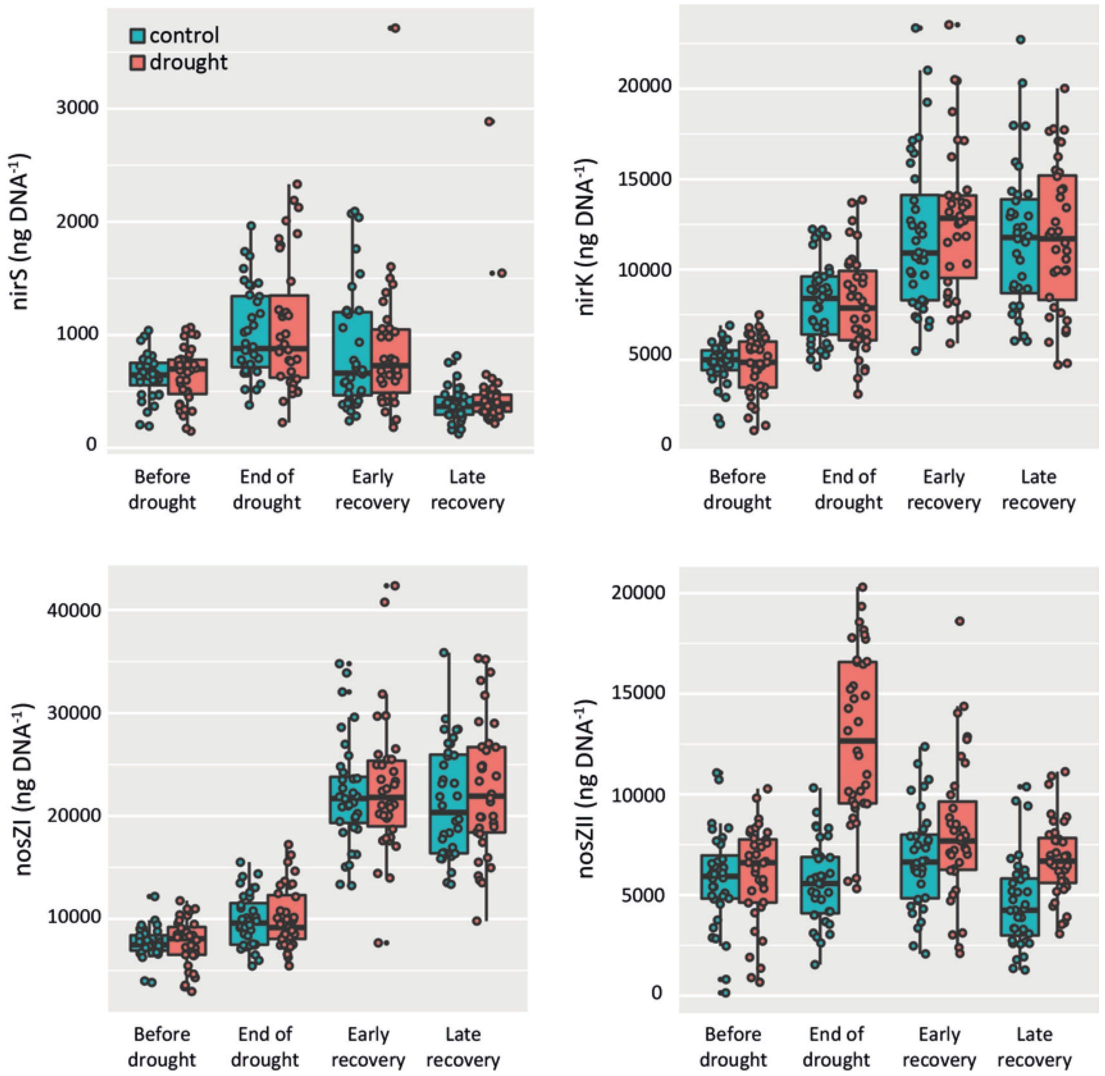


Supplementary Figure 7. Relative abundances of *nirS*, *nirK*, *nosZI* and *nosZII* genes as affected by drought over time. All genes were significantly affected by time (repeated measures ANOVA Sampling $P < 0.001$); only nosZII abundnaces were affected by drought, and this effect varied over time (Sampling x Drought interaction $F_{3,200} = 8.48$, $P < 0.001$). Lines in boxes represent median, top and bottom of boxes represent first and third quartiles, and whiskers represent 1.5 inter quartile range; dots represent single observations.

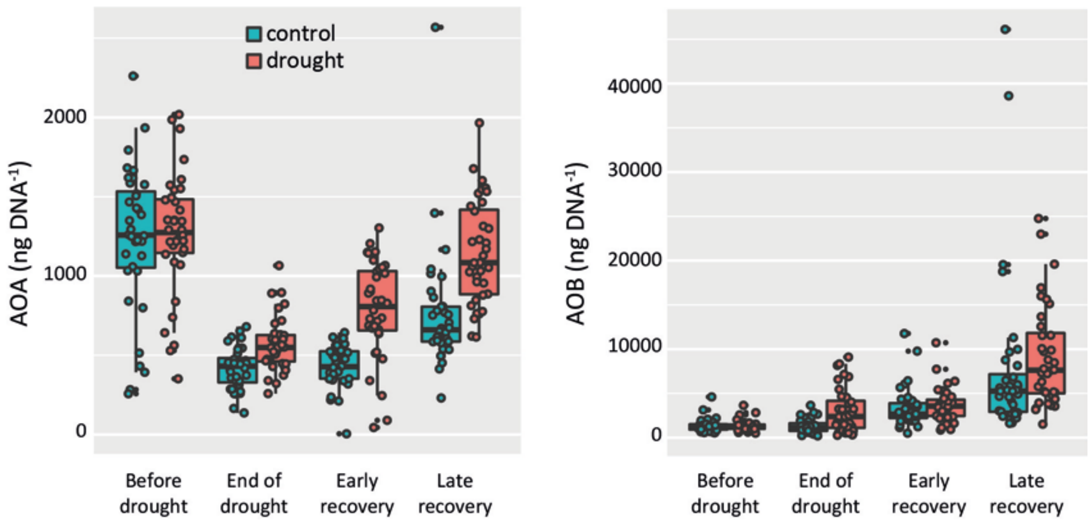


Supplementary Figure 8. Relative abundances of archaeal (AOA) and bacterial (AOB) *amoA* genes over time. Both genes were significantly affected by drought, and this effect varied over time (Sampling x Drought interaction $F_{3,202} = 3.77$, $P = 0.011$ and Sampling x Drought interaction $F_{3,202} = 3.38$, $P = 0.019$ for AOA and AOB respectively). Lines in boxes represent median, top and bottom of boxes represent first and third quartiles, and whiskers represent 1.5 inter quartile range; dots represent single observations.

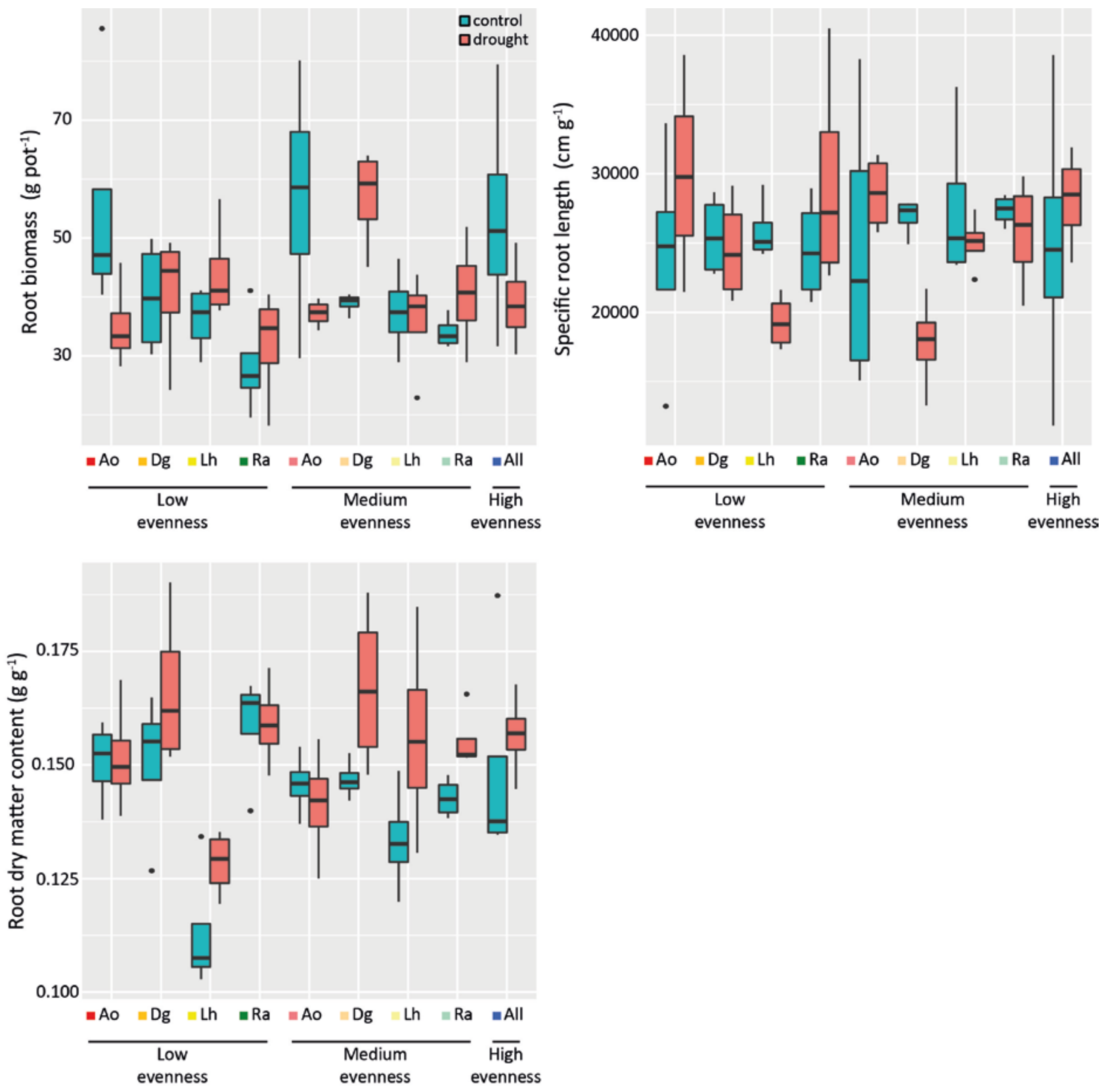


Supplementary Figure 9. Root biomass, specific root length, and root dry matter content per plant community treatment as affected by drought at the final (late recovery) sampling. The effect of drought on root biomass and specific root length depended on plant community treatment (Drought x Dominant species interaction $F_{4,48} = 4.81$, $P = 0.002$ and $F_{4,48} = 2.80$, $P = 0.036$ for root biomass and specific root length, respectively). Root dry matter content was increased by drought (Drought $F_{1,3} = 60.9$, $P = 0.004$). Lines in boxes represent median, top and bottom of boxes represent first and third quartiles, and whiskers represent 1.5 inter quartile range (n=4).

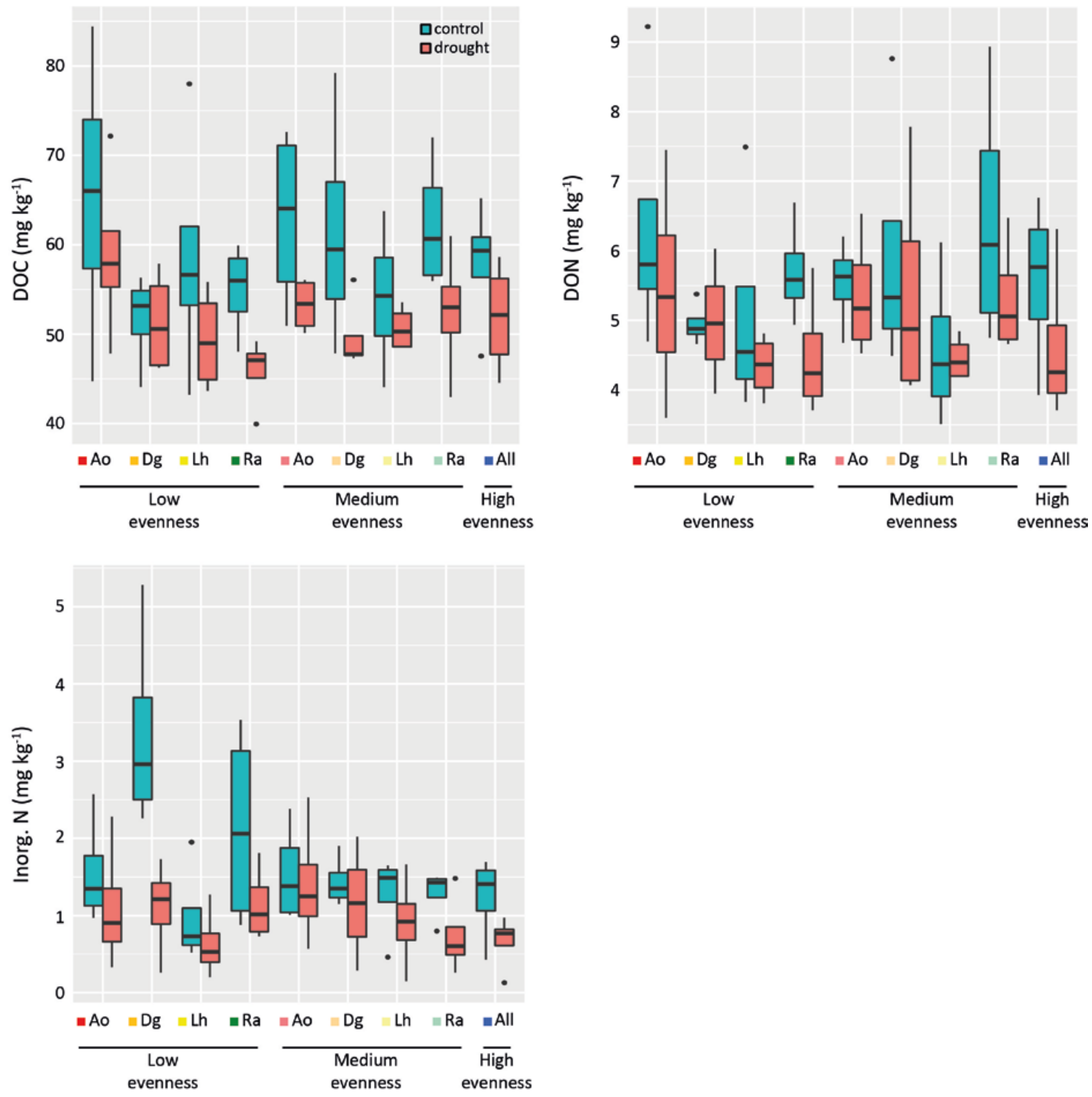


Supplementary Figure 10. Dissolved organic C (DOC), dissolved organic N (DON), and inorganic N per plant community treatment as affected by drought at the final (late recovery) sampling. All three properties were reduced by drought (Drought $F_{1,3}$ = 7.00, *P* = 0.078, $F_{1,3}$ = 13.8, *P* = 0.034, and $F_{1,3}$ = 21.4, *P* = 0.019, for DOC, DON, and inorganic N, respectively). Lines in boxes represent median, top and bottom of boxes represent first and third quartiles, and whiskers represent 1.5 inter quartile range (n=4).

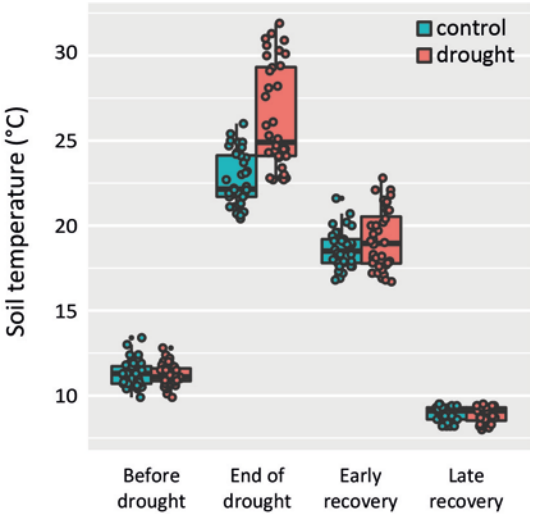


Supplementary Figure 11. Soil temperature as affected by drought over time. Soil temperature was increased by drought at the end of the drought (Sampling x Drought interaction $F_{3,200}$ = 37.9, $P < 0.001$). Lines in boxes represent median, top and bottom of boxes represent first and third quartiles, and whiskers represent 1.5 inter quartile range; dots represent single observations.

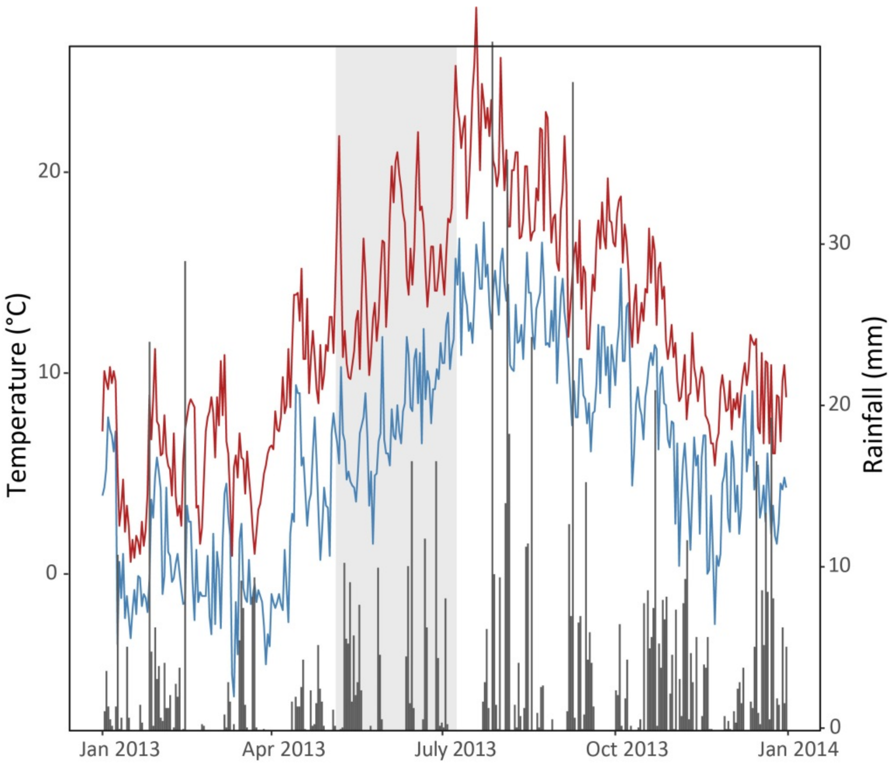


Supplementary Figure 12. Temperature and rainfall during the experimental period. Red lines indicate daily maximum temperature, blue lines indicate daily minimum temperature, grey bars are daily rainfall. The shaded area indicates the simulated drought.

Supplementary Table 1. Number of individuals of each species in the experimental plant communities. Each plant community contained all four species and a total number of 36 individual plants, but in varying abundances, resulting in four low evenness treatments, four medium evenness treatments, and one high evenness treatment. Each treatment was replicated four times.

| | **Low evenness** | | | | **Medium evenness** | | | | **High evenness** |
|---|---|---|---|---|---|---|---|---|---|
| Slow-growing grass *Anthoxanthum odoratum* | 30 | 2 | 2 | 2 | 18 | 6 | 6 | 6 | 9 |
| Fast-growing grass *Dactylis glomerata* | 2 | 30 | 2 | 2 | 6 | 18 | 6 | 6 | 9 |
| Slow growing herb *Leontodon hispidus* | 2 | 2 | 30 | 2 | 6 | 6 | 18 | 6 | 9 |
| Fast growing herb *Rumex acetosa* | 2 | 2 | 2 | 30 | 6 | 6 | 6 | 18 | 9 |
| Individuals/pot | 36 | 36 | 36 | 36 | 36 | 36 | 36 | 36 | 36 |

Supplementary Table 2. Network properties of bacterial and fungal co-occurrence networks (a) and of combined fungal-bacterial co-occurrence networks (b).

**a**

| | Bacterial network | | Fungal network | |
|---|---|---|---|---|
| | Control | Drought | Control | Drought |
| **Before drought** | | | | |
| Number of nodes | 831 | 839 | 113 | 88 |
| Number of edges | 1354 | 1452 | 274 | 244 |
| Clustering coefficient | 0.10 | 0.08 | 0.45 | 0.67 |
| R-square power law fit | 0.85 | 0.86 | 0.86 | 0.82 |
| **End of drought** | | | | |
| Number of nodes | 936 | 701 | 78 | 102 |
| Number of edges | 1552 | 978 | 112 | 138 |
| Clustering coefficient | 0.10 | 0.08 | 0.07 | 0.20 |
| R-square power law fit | 0.88 | 0.88 | 0.96 | 0.89 |
| **Early recovery** | | | | |
| Number of nodes | 637 | 369 | 141 | 102 |
| Number of edges | 1018 | 700 | 198 | 132 |
| Clustering coefficient | 0.19 | 0.24 | 0.18 | 0.12 |
| R-square power law fit | 0.94 | 0.91 | 0.85 | 0.97 |
| **Late recovery** | | | | |
| Number of nodes | 794 | 886 | 103 | 126 |
| Number of edges | 1267 | 2210 | 204 | 190 |
| Clustering coefficient | 0.09 | 0.17 | 0.36 | 0.22 |
| R-square power law fit | 0.91 | 0.93 | 0.98 | 0.86 |

**b**

| | Combined fungal-bacterial network | | | | | | |
|---|---|---|---|---|---|---|---|
| | Number of nodes | Number of edges | Proportion of bacterial nodes | Proportion of bacteria-bacteria edges | Proportion of fungi-fungi edges | Proportion of fungi-bacteria edges | Clustering coefficient |
| **Before drought** | | | | | | | |
| Control | 1313 | 2834 | 0.78 | 0.58 | 0.14 | 0.29 | 0.13 |
| Drought | 1246 | 2898 | 0.75 | 0.50 | 0.18 | 0.32 | 0.25 |
| **End of drought** | | | | | | | |
| Control | 1409 | 2736 | 0.75 | 0.57 | 0.13 | 0.30 | 0.10 |
| Drought | 1229 | 1990 | 0.71 | 0.49 | 0.16 | 0.35 | 0.11 |
| **Early recovery** | | | | | | | |
| Control | 1002 | 1712 | 0.74 | 0.59 | 0.12 | 0.29 | 0.16 |
| Drought | 641 | 1182 | 0.72 | 0.59 | 0.11 | 0.30 | 0.22 |
| **Late recovery** | | | | | | | |
| Control | 1332 | 2568 | 0.71 | 0.50 | 0.14 | 0.36 | 0.13 |
| Drought | 1546 | 5060 | 0.71 | 0.54 | 0.13 | 0.34 | 0.16 |

Supplementary Table 3. PCR primers and thermal cycling conditions used for quantification of different genes

| Genes and primers | Primer sequences (5' – 3') | Primer references | Thermal conditions[a] |
|---|---|---|---|
| **16S rRNA:**<br>341F<br>534R | <br>CCT ACG GGA GGC AGC AG<br>ATT ACC GCG GCT GCT GGC A | <br>Lopez-Gutierrez et al., 2004 | (95°C, 7 min) x 1<br>(95°C, 15 s; 60°C, 30 s; 72°C, 30 s; 80°C, 30 s) x 40<br>(95°C, 15 s;(60 to 95° C, 10 s, increment 0.5°)), x 1 |
| ***nirK*:**<br>nirK 876<br>nirK R3Cu | <br>ATY GGC GGV CAY GGC GA<br>GCC TCG ATC AGG TTR TGG TT | <br>Henry et al., 2006 | (95°C, 7 min) x 1<br>(95°C, 15 s; (63°C – 58°C, -1°/cycle), 30 s; 72°C, 30 s) x 6;<br>(95°C, 15 s; 58°C, 30 s; 72°C, 30 s; 80°C, 30 s) x 35<br>(95°C, 15 s;(60 to 95° C, 10 s, increment 0.5°)), x 1 |
| ***nirS*:**<br>nirSCd3aFm<br>nirSR3cdm | <br>AAC GYS AAG GAR ACS GG<br>GAS TTC GGR TGS GTC TTS AYG AA | <br>Throbäck et al., 2004 | (95°C, 7 min) x 1<br>(95°C, 15 s; (65°C – 60°C, -1°/cycle), 30 s; 72°C, 30 s) x 6;<br>(95°C, 15 s; 60°C, 30 s; 72°C, 30 s; 80°C, 30 s) x 35<br>(95°C, 15 s;(60 to 95° C, 10 s, increment 0.5°)), x 1 |
| ***nosZ*I:**<br>nosZ2F<br>nosZ2R | <br>CGC RAC GGC AAS AAG GTS MSS GT<br>CAK RTG CAK SGC RTG GCA GAA | <br>Henry et al., 2006 | (95°C, 7 min) x 1<br>(95°C, 15 s; (65°C – 60°C, -1°/cycle), 30 s; 72°C, 30 s) x 6;<br>(95°C, 15 s; 60°C, 30 s; 72°C, 30 s; 80°C, 30 s) x 35<br>(95°C, 15 s;(60 to 95° C, 10 s, increment 0.5°)), x 1 |
| ***nosZ*II:**<br>nosZII-F<br>nosZII-R | <br>CTIGGICCIYTKCAYAC<br>GCIGARCARAAITCBGTRC | <br>Jones et al., 2013 | (95°C, 7 min) x 1<br>(95°C, 15 s; 54°C, 30 s; 72°C, 30 s; 80°C, 30 s) x 40<br>(95°C, 15 s;(60 to 95° C, 10 s, increment 0.5°)), x 1 |
| **AOB *amoA*** | AmoA-1F (GGGGTTTCTACTGGTGGT) / AmoA-2R (CCCCTCKGSAAAGCCTTCTTC) | Rotthauwe *et al.*, 1997 | (95°C, 15 min) x1<br>(94°C, 15 s; 60 °C, 90 s; 80°C, 5 s) x 40<br>(95°C, 15 s;(60 to 95° C, 10 s, increment 0.5°)), x 1 |
| **AOA *amoA*** | Crenamo23F (ATGGTCTGGCTWAGACG) / Crenamo616R (GCCATCCATCTGTATGTCCA) | Tourna et al. (2008) | (95°C, 15 min) x1<br>(94°C, 15 s; 60°C, 60 s; 80°C, 5 s) x 40<br>(95°C, 15 s;(60 to 95° C, 10 s, increment 0.5°)), x 1 |

[a] Fluorescent signal was acquired at 80°C.

Supplementary Note 1.

We expected the observed increase in *D. glomerata* biomass to affect aboveground biomass both directly and indirectly through the change in plant community composition (see Figure 6 main paper). We then expected these changes in plant community composition to affect soil moisture and dissolved organic C and inorganic N.

We expected that soil moisture would affect all microbial community properties directly.

We hypothesised that bacterial and fungal communities would be directly affected by plant community composition, through mutualistic and antagonistic relationships, and indirectly, through the effects of plant community composition on soil moisture content and on DOC and inorganic N[1,2,3].

We expected bacterial as well as fungal community composition to influence the abundance of genes involved in the denitrification process[4].

We hypothesised that the abundance of *nir* genes would influence the abundance of *nosZ* genes, because they perform sequential steps in the denitrification process[4].

Supplementary Note 2.

We expected that drought would affect all microbial community properties and ecosystem processes directly through its effect on soil moisture[1].

We hypothesised that bacterial and fungal communities would be directly affected by plant community composition, through mutualistic and antagonistic relationships, and indirectly, through the effects of plant community composition on rates of photosynthesis (and thus belowground C inputs)[2] and soil moisture content[3].

We expected bacterial as well as fungal community composition to influence the abundance of genes involved in the denitrification process[4].

We hypothesised that the abundance of *nir* genes would influence the abundance of *nosZ* genes, because they perform sequential steps in the denitrification process[4].

Higher *nir* gene abundances increase $N_2O$ production through the production of NO, while higher *nosZ* gene abundances reduce N2O production because they consume $N_2O$[4].

We expected $N_2O$ production to be controlled by bacterial and fungal community composition directly, through their influence on soil nitrogen availability[5]. In addition, it is well known that rates of $N_2O$ production are controlled by soil moisture content and the availability of low molecular weight C compounds[4].

We expected $CO_2$ production (ecosystem respiration) to be directly controlled by the factors limiting heterotrophic and autotrophic respiration: soil moisture and photosynthesis, which provides C for both autotrophic and heterotrophic respiration. We also expected the composition of bacterial and fungal communities to influence $CO_2$ production[6].

Supplementary References


[1]De Vries, F. T. et al. Land use alters the resistance and resilience of soil food webs to drought. Nat. Clim. Change 2, 276-280 (2012).

[2]De Deyn, G. B., Quirk, H., Oakley, S., Ostle, N. & Bardgett, R. D. Rapid transfer of photosynthetic carbon through the plant-soil system in differently managed species-rich grasslands. Biogeosciences 8, 1131-1139, doi:10.5194/bg-8-1131-2011 (2011).

[3]Prevey, J. S. & Seastedt, T. R. Seasonality of precipitation interacts with exotic species to alter composition and phenology of a semi-arid grassland. J. Ecol. 102, 1549-1561, doi:10.1111/1365-2745.12320 (2014).

[4]Baggs, E. M. Soil microbial sources of nitrous oxide: recent advances in knowledge, emerging challenges and future direction. Curr. Opin. Env. Sust. 3, 321-327, doi:http://dx.doi.org/10.1016/j.cosust.2011.08.011 (2011).

[5]De Vries, F. T. et al. Extensive Management Promotes Plant and Microbial Nitrogen Retention in Temperate Grassland. PLoS ONE 7, e51201, doi:10.1371/journal.pone.0051201 (2012).

[6]Yuste, J. C. et al. Microbial soil respiration and its dependency on carbon inputs, soil temperature and moisture. Glob. Change Biol. 13, 2018-2035, doi:10.1111/j.1365-2486.2007.01415.x (2007).

[7]Lopez-Gutierrez J.C., Henry S., Hallet S., Martin-Laurent F., Catrou, G., Philippot L. Quantification of a novel group of nitrate-reducing bacteria in the environment by real-time PCR. J. Microbiol. Methods 57, 399-407 (2004).

[8]Henry S., Bru D., Stres B., Hallet S., Philippot, L. Quantitative detection of the *nosZ* gene, encoding nitrous oxide reductase, and comparison of the abundances of 16S rRNA, *narG, nirK*, and *nosZ* genes in soils. Appl. Env. Microbiol. 72, 5181-5189 (2006).

[9]Throbäck N., Enwall K., Jarvis A., Hallin, S. 2004. Reassessing PCR primers targeting *nirS*, *nirK* and *nosZ* genes for community surveys of denitrifying bacteria with DGGE. FEMS Microbiol. Ecol. 49, 401-417 (2004).

[10]Jones, C.M., Graf, D., Bru, D., Philippot, L., Hallin, S. The unaccounted yet abundant nitrous oxide reducing microbial community - a potential nitrous oxide sink. ISME J. 7, 417-426 (2013).

[11]Rotthauwe, J.H., Witzel, K.P. and Liesack, W. The ammonia monooxygenase structural gene amoA as a functional marker: molecular fine-scale analysis of natural ammonia-oxidizing populations. Appl. Env. Microbiol. 63:4704–4712 (1997).

[12]Tourna, M., Freitag, T.E., Nicol, G.W. and Prosser, J.I.. Growth activity and temperature responses of ammonia- oxidizing archaea and bacteria in soil microcosms. Env. Microbiol. 10, 1357–1364 (2008).
