# Supplementary material for: Soil bacterial networks are less stable under drought than fungal networks
Source: Nat Commun. 2018 Aug 2;9:3033. doi: 10.1038/s41467-018-05516-7 (PMC6072794; doi:10.1038/s41467-018-05516-7)
Supplement: Supplementary file 3 — Description of Additional Supplementary Files [file 41467_2018_5516_MOESM3_ESM.pdf]

Description of Additional Supplementary Files

File Name: Supplementary Data 1
Description: Node properties for bacterial networks. Includes node-level network statistics (norm_degree and betweenness), network-level statistics (clustering_coeff, clustering_coeff_rand, cluster_ratio), and indicator analysis results for each node (ind [n, d, or c], mean_drought, mean_control, mean_tot, indval, indval_drought, indval_control, p, fold.change, mean.diff, fold.change.log).

File Name: Supplementary Data 2
Description: Node properties for fungal networks. Includes node-level network statistics (norm_degree and betweenness), network-level statistics (clustering_coeff, clustering_coeff_rand, cluster_ratio), and indicator analysis results for each node (ind [n, d, or c], mean_drought, mean_control, mean_tot, indval, indval_drought, indval_control, p, fold.change, mean.diff, fold.change.log).
